# Silibinin Anticancer Effects Through the Modulation of the Tumor Immune Microenvironment in Triple-Negative Breast Cancer

**DOI:** 10.3390/ijms26136265

**Published:** 2025-06-28

**Authors:** Shubham D. Mishra, Patricia Mendonca, Sukhmandeep Kaur, Karam F. A. Soliman

**Affiliations:** 1 Division of Pharmaceutical Sciences, College of Pharmacy and Pharmaceutical Sciences, Institute of Public Health, Florida A&M University, Tallahassee, FL 32307, USA; shubham1.mishra@famu.edu (S.D.M.); sukhmandeep1.kaur@famu.edu (S.K.); 2 Department of Biology, College of Science and Technology, Florida A&M University, Tallahassee, FL 32307, USA

**Keywords:** silibinin, flavonoids, breast cancer, TNBC, PD-L1/PD-1, Nrf2, CCL2, tumor immune microenvironment, JAK/STAT3, MUC1-C

## Abstract

Triple-negative breast cancer (TNBC), characterized by the absence of estrogen receptor (ER), progesterone receptor (PR), and human epidermal growth factor receptor 2 (HER2), remains a therapeutic challenge due to its aggressive nature, limited treatment options, and high recurrence rates. Current therapies, including chemotherapy and immune checkpoint inhibitors, face resistance driven by tumor heterogeneity, immunosuppressive signaling, and dysregulated redox pathways. This review explores silibinin’s potential to modulate the tumor immune microenvironment (TIME) and overcome therapeutic resistance in TNBC. Silibinin exerts multifaceted anticancer effects by suppressing PD-L1 expression through the inhibition of JAK/STAT3 signaling and MUC1-C interaction, attenuating NF-κB-driven inflammation, and downregulating CCL2-mediated recruitment of tumor-associated macrophages (TAMs). Additionally, silibinin disrupts redox adaptation by targeting the Nrf2-EGFR-MYC-TXNIP axis, enhancing oxidative stress and chemosensitivity. Preclinical studies highlight its ability to inhibit epithelial–mesenchymal transition (EMT), reduce cancer stem cell (CSC) populations, and synergize with existing therapies like PD-1 inhibitors. Despite its low bioavailability, advanced formulations such as liposomes and nanoparticles show promise in improving delivery and efficacy. By reshaping TIME through dual antioxidant and immunomodulatory mechanisms, silibinin emerges as a viable adjunct therapy to reverse immunosuppression and chemoresistance in TNBC.

## 1. Introduction

Triple-negative breast cancer (TNBC) is an aggressive subtype of breast cancer distinguished by the lack of expression of ER (estrogen receptors), PR (progesterone receptors), and HER2 (human epidermal growth factor receptor 2). Due to its unique molecular signature, TNBC is resistant to conventional hormone and HER2-targeted therapies, leaving chemotherapy as the primary treatment option. Unfortunately, chemotherapy has presented abominably high recurrence rates and toxicity to patients [1]. The American Cancer Society (ACS) reports that breast cancer is the second major cause of death, affecting women all over the globe [2]. In 2024, the projected mortalities due to breast cancer in the United States were 42,250 women, and most of these deaths would be in TNBC [2]. TNBC causes approximately 10–15% of all breast cancer diagnoses yet it disproportionately accounts for breast cancer deaths due to its aggressive nature and limited treatment possibilities [3]. Metastatic TNBC prognosis is dreadful, with a survival rate of 12 percent for five years. Therefore, there is an urgent need for improved and targeted treatment alternatives [4].

**Figure 1:**
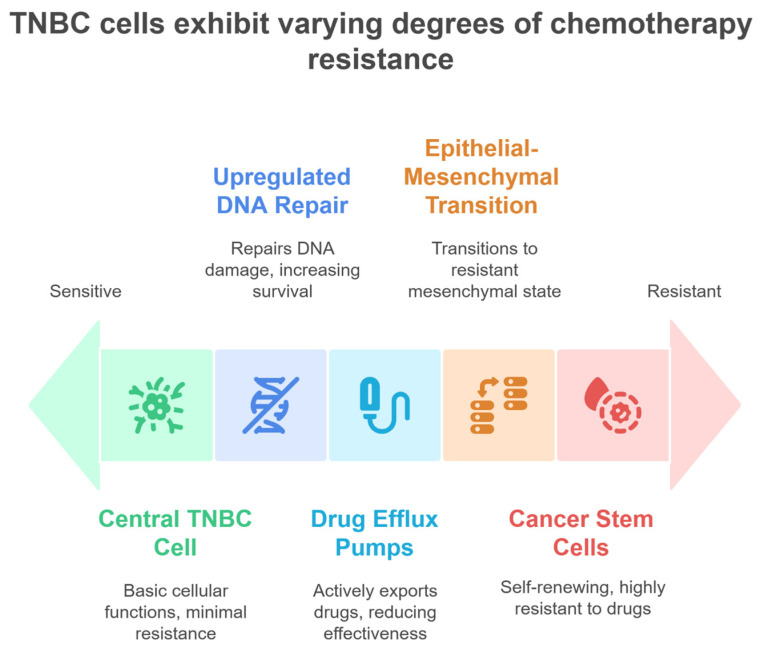
A graphical summary of the key mechanisms underlying chemotherapy resistance in TNBC, including the role of epithelial–mesenchymal transition (EMT), drug efflux pumps, cancer stem cell enrichment, and DNA repair pathways.

Most of the current treatments for TNBC consist of chemotherapies, often given in combination with surgery and radiation therapy. While chemotherapy might initially reduce the tumor’s size, its major effects are somewhat limited, with significant side effects. Despite its efficacy, chemotherapy in TNBC is often limited by notable side effects. These include severe fatigue, nausea, vomiting, alopecia, and myelosuppression leading to neutropenia and an increased risk of infections. Additionally, anthracyclines may cause cardiotoxicity, and taxanes can induce peripheral neuropathy, all of which impact patient quality of life and treatment continuity [5]. The inability to entirely prevent recurrence has often led to the development of drug resistance, undermining long-term treatment success (Figure 1) [6]. Modern treatments are recognized to work well for specific subgroups of TNBC patients, but generally, they still have severe side effects for the patient [7]. The lack of multiple therapeutic options with specific targets, combined with the high heterogeneity and aggressiveness of TNBC, emphasizes the great need for developing novel strategies that can overcome these disadvantages with minimal toxicity to patients [1].

Over the past several years, and notably during 2024–2025, a growing body of scholarly literature has highlighted the potential of natural compounds and immunotherapeutic strategies in overcoming the inherent challenges associated with TNBC. Research in *Biomarker Studies* in 2024 demonstrated that the use of natural agents in combination with immune checkpoint inhibitors enhances antitumor activity by modulating key signaling pathways, including PD-L1, NF-κB, and Nrf2 [8]. In addition, a recent review in *Molecules* in 2025 highlighted that a range of bioactive natural compounds, including flavonoids, can act synergistically with immunotherapies in preclinical models of TNBC to augment the efficacy of conventional treatment protocols [9]. Additionally, a study published in *Frontiers in Genetics* in 2024 confirmed that targeting both cancerous cells and components of the tumor immune microenvironment can augment T-cell cytolytic functions, further supporting the hypothesis that natural compounds can alter the immunosuppressive environment typically associated with TNBC [10].

Natural compounds have been receiving considerable research interest in recent years because they target various pathways implicated in cancer development with reduced toxicity [11]. Among naturally occurring compounds, flavonoids represent a diverse class of polyphenolic substances widely found in fruits, vegetables, and medicinal plants. These compounds have garnered considerable attention due to their biological activity spectrum, including antioxidative, anti-inflammatory, and anti-cancer effects [12]. They work through multiple pathways to influence critical cellular events such as cell growth, apoptosis, and the invasiveness of the cancer cells [11]. Flavonoids are particularly important because they have been found to target cancer cells while leaving the normal ones relatively unaffected, thereby increasing their suitability in cancer therapy [13]. Their ability to strengthen standard treatment and mitigate treatment-induced side effects also positions them as promising adjunct or alternative therapies for TNBC [12]. Furthermore, emerging evidence suggests that other flavonoids may also be promising. A very recent study by Rana and Mumtaz (2025) demonstrated that prunin effectively modulates key oncogenic pathways and remodels the tumor microenvironment, thereby broadening the scope of flavonoid-based therapeutic strategies in TNBC [14]. Specifically, prunin was shown to suppress the PI3K/AKT signaling cascade, leading to reduced tumor cell proliferation and enhanced apoptotic activity. It also downregulated pro-inflammatory cytokines and altered macrophage polarization within the tumor milieu, indicating a capacity to reverse immunosuppression [14].

Among flavonoids, silibinin has been extensively studied for its anticancer potential and has demonstrated excellent potential in TNBC preclinical models [15]. Silibinin acts on various mechanisms, as tumor cell proliferation inhibition, apoptosis induction, and tumor dissemination inhibition [16]. Silibinin is also implicated in making malignant cells more sensitive to cytotoxic chemotherapy drugs, one approach toward reversing drug resistance, which is a significant concern in therapies for TNBC patients [15]. Thus, silibinin’s antioxidant and anti-inflammatory properties may counteract some of the adverse effects of chemotherapy and enhance patient response [17]. However, despite the progress already made, the complete therapeutic profile of silibinin and other flavonoids in TNBC remains to be further investigated, particularly in clinical contexts. Additional research is needed to elucidate their molecular mechanisms, enhance their bioavailability, and establish their effectiveness with current treatment regimens [15].

This review describes current progress in using silibinin as an anticancer and chemopreventive agent in human breast cancer, referencing various “in vitro” and “in vivo” studies and clinical trials that include silibinin in treatment regimens. Moreover, the review also discusses the potential implications of silibinin in inhibiting epigenetic modifications associated with breast cancer resistance [18]. In addition, limitations regarding solubility, stability, and low bioavailability of silibinin were also discussed in relation to the present strategies adopted to enhance its therapeutic activity [19]. Thus, this review aims to provide an overview of current research supporting the therapeutic application of silibinin for TNBC by modulating the tumor immune microenvironment (TIME).

## 2. Breast Cancer

Breast cancer remains one of the most prevalent cancers worldwide, with significant implications for public health. According to the American Cancer Society, breast cancer led the incidence of almost 31% of all new cancer cases among women in 2024, with an estimated 297,790 new diagnoses and 43,170 deaths in the United States alone [2]. Heterogeneity is quite enormous, and differences depend on factors such as hormone receptor status, genetic alterations, and changes in the tumor microenvironment (TIME). Of all the subtypes of breast cancer, the most aggressive one is TNBC, which confers a much poorer prognosis, higher rates of recurrence, and fewer treatment options compared to hormone receptor-positive or HER2-enriched subtypes [1,4].

### 2.1. Triple-Negative Breast Cancer (TNBC)

TNBC is a subtype of breast cancer distinguished by the lack of expression of estrogen receptors (ERs), progesterone receptors (PRs), and human epidermal growth factor receptor 2 (HER2) [6]. According to Lehmann et al. (2011), using expression profile analyses, TNBC patients were found to have six different molecular subtypes, namely basal-like 1 (BL1), BL2 (basal-like 2), immunomodulatory (IM), mesenchymal (M), mesenchymal stem-like (MSL), and luminal androgen receptor (LAR) [3]. These subtypes exhibit distinct genetic profiles and therapeutic susceptibility. In general, BL1 tumors have enhanced sensitivity to platinum-based chemotherapy through DNA damage response pathways, while LAR tumors express androgen receptors and could be treated with anti-androgenic therapies [3]. In more recent studies, further refinement was made about the classification of TNBC into either the basal-like immune-activated (BLIA) or the basal-like immunosuppressed (BLIS) subtypes, distinguished by their level of immune infiltration and the expression of different checkpoint molecules [20]. BLIA tumors show elevated STAT signaling and programmed death-ligand 1 (PD-L1) expression, whereas immunosuppressive markers like VTCN1 characterize BLIS tumors [21].

Moreover, the Fudan University classification (FUSCC) system integrates mRNA and long non-coding RNA (lncRNA) profiles, categorizing TNBC into four subtypes: IM, LAR, M, and BLIS [22]. Infiltration of immune cells into IM tumors characterizes them, and they show a better response to immunotherapy, while BLIS tumors carry stromal activation and resist conventional treatment altogether [22]. As for prognosis, BL2 and M subtypes correlate with poor survival, while LAR tumors tend to have longer progression-free survival (PFS) since they are dependent on androgen signaling pathways. It signifies that specific therapies targeted exclusively for certain types of TNBC will be needed to improve the quality of TNBC patients’ lives [3,6].

### 2.2. Racial Disparities in TNBC Incidence and Outcomes

The impact of TNBC on African American women, in comparison with Caucasian American women, is seen to have a two-fold increase in incidence and 40% greater death rates; this after taking into consideration the socio-economic factors [4]. Genetic susceptibilities, including variable frequencies of BRCA1 mutations and ancestral tumor biology, further heighten this disparity [7]. Tumors in African American patients frequently exhibit elevated expression of pro-inflammatory cytokines (e.g., IL-6) and immune checkpoint molecules like PD-L1, which further drives aggressive phenotypes and limits therapeutic responses [22,23]. In addition, system-wide inequities in healthcare access, such as delayed diagnosis and utilization of genetic testing, worsen health outcomes [4]. Recent studies on African American women report that alterations in TIME include increased infiltration of myeloid-derived suppressor cells (MDSCs), which can lead to immune suppression. MDSCs suppress anti-suitor immunity and foster chemoresistance. To eliminate these differences, health screening, justifiable population selection for intervention trials, and treatment that targets molecular differences specific to race are needed [24].

### 2.3. Current Therapeutic Strategies for TNBC

#### 2.3.1. Chemotherapy and Radiotherapy

The treatment of TNBC is primarily based on chemotherapy due to the lack of targetable receptors. Anthracycline–taxane regimens are commonly used in neoadjuvant settings to shrink tumors before surgery, with pathological complete response (PCR) rates correlating with improved survival [7]. Platinum agents like carboplatin are prioritized for BRCA-mutated TNBC due to synthetic lethality with DNA repair deficiencies [25]. Radiotherapy is also employed post-lumpectomy to reduce local recurrence, though its efficacy in metastatic TNBC is limited [26]. Despite initial responses, chemoresistance often arises from mechanisms such as the upregulation of drug efflux pumps (e.g., ABC transporters), activation of pro-survival pathways (e.g., nuclear factor kappa B (NF-κB)), and epithelial–mesenchymal transition (EMT) [24,27].

#### 2.3.2. Immunotherapy

**Figure 2:**
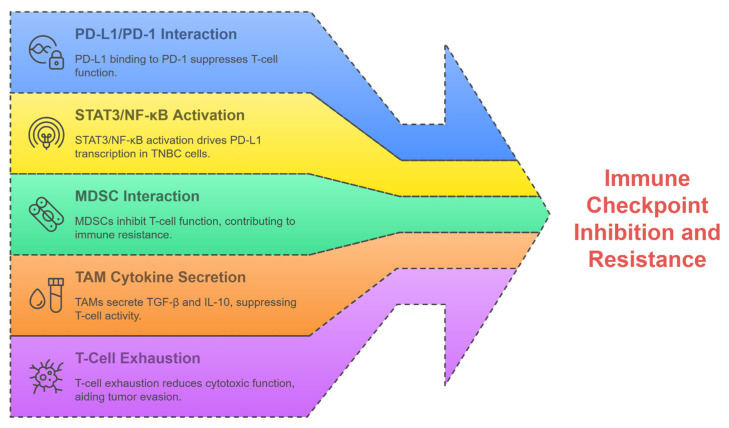
A visual representation of immune checkpoint inhibition and resistance in TNBC, highlighting PD-L1/PD-1 interaction, recruitment of myeloid-derived suppressor cells (MDSCs), and T-cell exhaustion.

Immune checkpoint inhibitors (ICIs) block the PD-1/PD-L1 interaction, revolutionizing the treatment of TNBC. The IMpassion130 trial proved that the combination of atezolizumab and nab-paclitaxel resulted in an improved progression-free survival in PD-L1-positive metastatic TNBC, which eventually led to an FDA approval [7]. However, only 20–40% of patients respond to treatment, and acquired resistance often develops due to T-cell exhaustion (Figure 2), upregulation of alternative checkpoints (e.g., LAG-3, TIM-3), and immunosuppressive TIME components like regulatory T cells (Tregs) [28].

Immunotherapy resistance often stems from adaptive immune evasion mechanisms, including PD-L1 modulation, secretion of immunosuppressive cytokines (e.g., TGF-β, IL-10), and recruitment of MDSCs (Figure 2) [24,29]. Recently, several studies have pointed out how TIME reprogramming and restoration of therapeutic sensitivity could be achieved via targeting transcription factors like STAT3 and NF-κB [30,31]. Strategies to overcome resistance involve combining ICIs with natural compounds, such as silibinin, to enhance immunogenicity and targeting stromal interactions, e.g., the CCL2/CCR2 axis and decreased tumor-associated macrophage (TAM) recruitment [22,32].

### 2.4. Resistance in TNBC Therapies

The chemotherapy resistance of TNBC occurs through various molecular mechanisms, many of which are due to tumor heterogeneity and adaptive signaling pathways involved. Adaptive activation of NF-κB and STAT3 pathways promotes cell survival and drug efflux, while EMT enhances metastatic potential and stemness [24,27]. The NF-κB transcription factor is known to induce anti-apoptotic proteins such as Bcl-2 and XIAP [27,33], whereas STAT3 is linked to the upregulation of multidrug resistance genes like MDR1 (ABCB1) [34,35]. In addition, EMT regulators such as Snail, Twist, and ZEB1 repress the transcription of E-cadherin, which enables mesenchymal transition, thereby inducing CSC-like properties and contributing significantly to resistance to treatment [22,36]. Genomic and transcriptomic heterogeneity, as identified by Burstein et al. in 2015 and Bareche et al. in 2018, are responsible for divergent responses to cytotoxic agents, allowing subpopulations of resistant cancer cells to survive [20,21]. TAMs further exacerbate chemoresistance by secreting CCL2, which activates AKT/β-catenin signaling to promote EMT and cancer stem cell (CSC) enrichment, as demonstrated by Chen et al. (2022) [22]. These CSCs exhibit intrinsic resistance due to enhanced DNA repair and drug efflux mechanisms. Additionally, the hyperactivation of survival pathways such as PI3K/AKT/mTOR enhances cell proliferation and reduces apoptosis, further limiting chemotherapy efficacy [37].

Radiotherapy resistance in TNBC is linked to the TIME dynamics and cellular plasticity. Specifically, cellular plasticity enables reversible phenotypic transitions, such as radiation-induced dedifferentiation into CSCs with enhanced DNA repair capacity, while dynamic TIME alterations (e.g., post-radiation surges in TGF-β or IL-6) promote immunosuppressive fibroblast activation and T-cell exhaustion [22,26,38]. Hypoxia, a common feature of TNBC, induces metabolic reprogramming and CSC maintenance, which are associated with radioresistance [38]. CSCs, characterized by their self-renewal capacity and upregulated DNA damage response pathways, evade radiation-induced cell death [22]. Furthermore, EMT-driven phenotypic plasticity enables tumor cells to adopt a mesenchymal state, which confers resistance to ionizing radiation by enhancing their migratory potential and reducing their vulnerability to oxidative stress. These adaptive mechanisms are compounded by the TIME’s role in shielding tumor cells through stromal interactions and immunosuppressive signals [22].

Immunotherapy resistance in TNBC arises from both tumor-intrinsic and immune-related factors. While PD-L1 expression is a biomarker for ICI response, its heterogeneous distribution in TNBC tumors limits consistent therapeutic efficacy with the development of resistance [39]. Intrinsic PD-1/PD-L1 inhibitor resistance is also associated with elevated CCL2 secretion, which recruits immunosuppressive TAMs and inhibits cytotoxic T-cell infiltration [22,29]. CD8+ T-cell dysfunction, driven by IL-6 signaling within the TIME, further impairs antitumor immunity and promotes ICI resistance [28]. Moreover, tumor cell-intrinsic CD28 signaling has been shown to suppress PD-L1 expression, thereby reducing antigen presentation and promoting immune evasion. These mechanisms interrelate to define the complex nature of immunotherapy resistance in TNBC [40].

Overlapping pathways mediate both chemoresistance and immunotherapy resistance in TIME. CCL2/AKT/β-catenin signaling drives chemoresistance via CSC expansion and recruits immunosuppressive myeloid cells, blunting adaptive immune responses [29,41]. Similarly, PI3K/AKT/mTOR activation, which promotes tumor cell survival during chemotherapy, concurrently upregulates immunosuppressive cytokines such as IL-6, thereby fostering a hostile tumor microenvironment (TIME) to T-cell activity. Such crosstalk between resistance mechanisms highlights the need for comprehensive molecular profiling to address multifactorial therapeutic barriers [28,37].

In addition, epigenetic modifications and post-translational regulation further entrench resistance across treatment modalities. Transglutaminase 2 (TGM2)-induced PD-L1 stabilization in TNBC cells creates an immune-suppressive niche, rendering ICIs ineffective even in PD-L1-positive tumors [29]. Additionally, metabolic adaptations such as increased lactate production in hypoxic regions acidify the TIME, impairing both chemotherapeutic drug activity and T-cell function [38]. These findings emphasize the role of dynamic TIME interactions in sustaining resistance to conventional and immunotherapeutic agents [29].

The basis for TNBC resistance to chemotherapy, radiotherapy, and immunotherapy lies in genetic diversity, the persistence of stem cell-like cells, and immunosuppressive signaling. The TIME acts as a point for all these processes, where hypoxia, cytokines, and stroma collectively disrupt the therapeutic effects. Without targeting these multifaceted mechanisms, overcoming resistance in TNBC remains a significant clinical challenge [28,38].

## 3. Flavonoids Effects on Breast Cancer Tumor Immune Microenvironment

Flavonoids are a variety of polyphenolic structures naturally found in fruits, vegetables, grains, and medicinal herbs. They can be subdivided into several groups, like flavonols, flavones, flavanones, isoflavones, and anthocyanins, each with specific chemical structures and biological activities [11,42]. These compounds are widely recognized for their antioxidant, anti-inflammatory, and chemopreventive properties, making them promising candidates for cancer prevention and therapy. They exert their anticancer effects through free radical scavenging, modulation of multiple signaling pathways, induction of apoptosis, and blockage of angiogenesis and metastasis in several types of cancer [11,43].

Combining flavonoids with chemo or radiotherapy has shown enhanced antitumor immune responses [44]. Li et al. (2023) demonstrated that flavonoids synergized with paclitaxel to reduce tumor burden in mice by elevating IFN-γ and granzyme B levels, markers of activated CD8+ T-cells [45]. Similarly, Han et al. (2021) found that flavonoid-loaded nano-complexes improved the efficacy of anti-HER2 therapies by depleting M2 macrophages and enhancing antibody-dependent cellular cytotoxicity [46]. Wang et al. (2024) also noted that flavonoid sensitized TNBC cells to doxorubicin by suppressing STAT3-mediated survival signals [47]. Flavonoid conditioning of TIME makes it more receptive to conventional therapies while dampening rebound immunosuppression effects [45,46,47].

### 3.1. Silibinin: Chemical Structure and Classification

**Figure 3:**
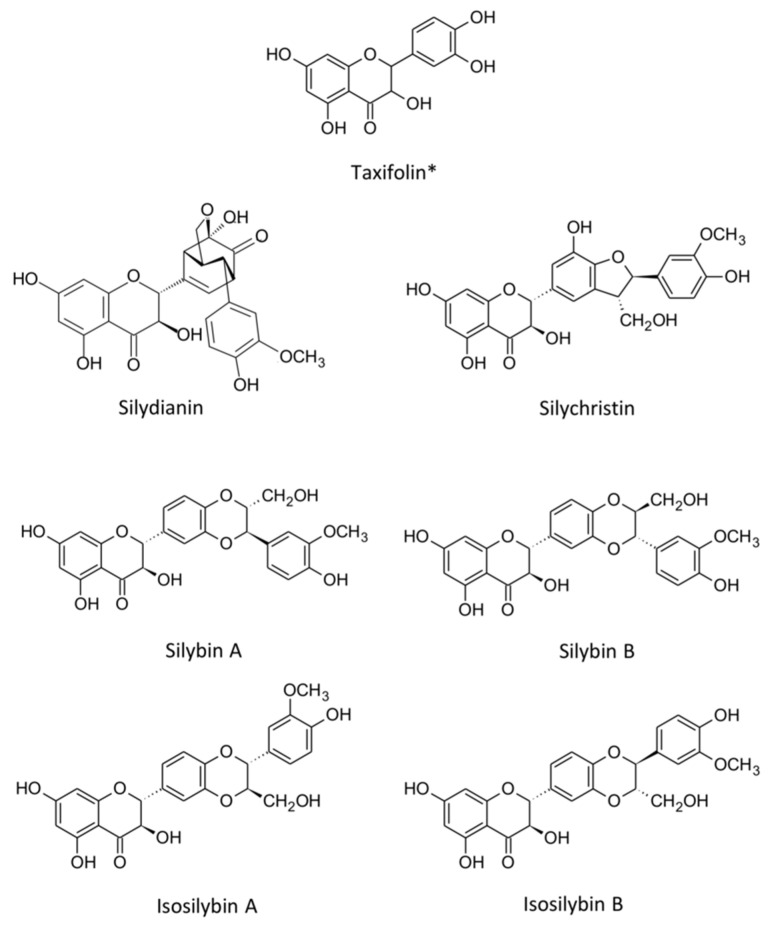
This figure illustrates the chemical structure of silibinin, a flavonolignan from *Silybum marianum*, along with its major derivatives (e.g., silychristine, silydianin, isosilibinin A and B) and the related flavonoid taxifolin. Silibinin is composed of a polyhydroxylated flavonoid moiety (structurally similar to taxifolin) attached to a lignan unit. The flavonoid segment, rich in hydroxyl groups, is critical for antioxidant properties and free radical scavenging, which help mitigate oxidative stress in cancer cells. The additional lignan portion is believed to enhance lipophilicity and cellular uptake, thereby broadening the spectrum of oncogenic signaling pathways (e.g., NF-κB, STAT3, and Nrf2) that can be modulated. In contrast, taxifolin, lacking the lignan moiety, may exhibit differences in bioavailability and bioactivity. These structural variations among silibinin and its derivatives compared to taxifolin underline how modifications in hydroxylation and overall molecular architecture can influence their anticancer efficacy [51]. * = Taxifolin is a precursor in the biosynthesis of silibinin.

Silibinin or silybin is a flavonolignan (Figure 3), a subclass of flavonoids, found in the milk thistle plant’s seeds (*Silybum marianum* L.). It is the primary active constituent of silymarin, a complex mixture of flavonolignans that includes silychristine, silydianin, and isosilibinin A and B [48,49]. Chemically, silybin A and silybin B are found in approximately equal proportions and are the main constituents of silymarin. Silibinin comprises two central units: a flavonoid moiety (taxifolin) and a lignan moiety (coniferyl alcohol). Its molecular formula is C_25_H_22_O_10_, with a molecular weight of 482.44 g/mol [50]. The molecular structure of silibinin comprises a conjugated system and multiple hydroxy groups, which collectively contribute to silibinin’s antioxidant properties and enable it to interact with cellular targets [50]. Due to the unique chemical structure of silibinin, which allows it to modulate multiple signaling pathways in cancer progression, it is a highly potent chemopreventive agent [48,50].

### 3.2. Silibinin Bioavailability

The bioavailability of silibinin is approximately 23–47% when administered orally, primarily due to restricted absorption from the gastrointestinal tract and substantial first-pass metabolism by the liver [52,53]. The low solubility of silibinin in water further complicates its delivery, as it tends to form aggregates in aqueous solutions, reducing its absorption efficiency [54]. Due to these difficulties, researchers have developed an interest in creating various formulations for silibinin, including nanoparticles, liposomes, and phospholipid complexes, to enhance the solubility and bioavailability of silibinin [53,54]. El-Samaligy et al. (2006) demonstrated that liposome-encapsulated silibinin exhibited improved stability and bioavailability, resulting in a consequent enhancement of the anti-cancer effects observed in preclinical models [54]. Likewise, silibinin-phospholipid complexes have been shown to enhance the absorption and therapeutic efficacy of the drug, potentially leading to clinical applications [53]. These advancements showed promising outcomes in preclinical studies by increasing silibinin delivery to the cancer cells and enhancing its chemopreventive activities [50].

Recently, developments with nanoparticle-based carriers, including those developed for curcumin analogs, provide outlines for pharmacokinetic enhancement for silibinin [55]. Encapsulation in a liposome or phospholipid complex is advantageous for solubilization and tissue-selective delivery, thereby prolonging exposure to chemopreventive concentrations. Lashgarian et al. (2020) have shown that silibinin is anti-migratory in a dose-dependent fashion, indicating a great need for formulations aimed at maintaining therapeutic thresholds in target tissues [56]. Notwithstanding, these innovations are significant for translating the preclinical efficacy of silibinin into clinical scenarios with special concentrated efforts in high-risk populations with apparently fewer preventive options [57].

### 3.3. Silibinin Toxicity

In terms of toxicity, while several preclinical and clinical studies have been published reporting minimal adverse effects, silibinin is considered relatively safe and well-tolerated [58]. Animal studies on acute or chronic silibinin toxicity confirm the highly safe profile, with no significant organ toxicity or mortality observed even at high doses [58,59]. Singh and Agarwal (2005) showed no significant toxicity signals in rats that received silibinin in doses up to 2000 mg/kg body weight, highlighting its potential for safe use in humans [58]. In human studies, silibinin in doses ranging from 140 to 700 mg/day presented no side effects [59]. Common mild side effects include gastrointestinal discomfort, such as nausea and diarrhea, which are typically transient and resolve without intervention [59,60]. Given the low toxicity profile of silibinin and its strong chemopreventive activity, it is a good candidate for further studies as an effective therapeutic agent in TNBC [58,61]. The use of silibinin for a long time in conventional medicine for treating liver disorders shows that the safety of silibinin is supported, with little to no reports of serious adverse events [60].

### 3.4. Silibinin Pharmacological Effects on Multiple Cancer Types

Silibinin exhibits significant anticancer effects against several malignancies, with substantial evidence supporting its efficacy both preclinically and clinically. In prostate cancer, silibinin inhibits integrin signaling, thereby reducing fibronectin-induced motility and invasiveness [50]. It degrades androgen receptors via the PI3K-Akt-Mdm2 pathway, inhibiting tumor growth and sensitizing cells to apoptosis [27,49]. Clinical trials, such as those using high-dose silibinin–phytosome before prostatectomy, show reduced prostate-specific antigen (PSA) levels and enhanced therapeutic outcomes [59,62].

For hepatocellular carcinoma (HCC), silibinin induces apoptosis by downregulating TGFα-EGFR autocrine loops and inhibiting survival pathways [16,17]. It also suppresses constitutive TGF-EGFR signaling, reducing tumorigenicity and metastatic potential in HCC models [16]. Silibinin has been shown to counteract fibronectin-mediated survival mechanisms and to work synergistically with chemotherapeutic agents to stop metastasis in lung cancer [18]. Additionally, it alters mitochondrial dynamics to promote mitochondrial fusion for blocking cancer cell migration [63].

In skin cancer, silibinin prevents UV-induced DNA damage by scavenging ROS and modulating MAPK/NF-κB pathways, significantly reducing tumor incidence [58,64]. The antioxidant qualities help protect against oxidative stress, while its anti-inflammatory effects suppress pro-tumorigenic signaling [27,58]. Silibinin also shows promise in pancreatic cancer, where it targets MUC1-C oncoprotein to inhibit HIF-1α-driven metabolic reprogramming, thereby enhancing sensitivity to gemcitabine and radiotherapy [65,66].

Recent evidence indicates that silibinin may play a role in colon cancer by downregulating the expression of PD-L1 via modulation of Nrf2 to counteract oxaliplatin resistance and minimize immune evasion [67]. Furthermore, silibinin induced ferroptosis in osteosarcoma in DNA-PKcs/AKT/Nrf2 pathways, enhancing cisplatin sensitivity while countering PD-L1-mediated immune escape [68]. This is strengthened even more by hybrid forms that enhance bioavailability and therapeutic efficacy in preclinical models, such as liposome-encapsulated silibinin [54]. Overall, silibinin acting against oncogenic signaling, metabolic reprogramming, and immune evasion truly indicates it can act across a variety of cancers in a multidimensional capacity as a chemopreventive or adjunct therapeutic factor [48,61].

### 3.5. Silibinin Chemopreventive Effect on Triple-Negative Breast Cancer

Silibinin’s chemopreventive potential in TNBC is rooted in its ability to elevate oxidative stress, inflammation, and metastatic pathways, mirroring mechanisms observed in other phytochemicals. Specifically, it elevates oxidative stress by suppressing antioxidant enzymes (e.g., SOD, catalase) and depleting glutathione; amplifies inflammation through NF-κB/STAT3-driven cytokine surges (e.g., IL-6, TNF-α); and activates metastatic pathways via EMT transcription factors (e.g., Snail, Twist) and matrix metalloproteinases (MMPs) [15,27,69]. Like anthocyanins, which suppress TNBC invasion by downregulating Akt/mTOR signaling and activating apoptosis [70]. Silibinin inhibits metastasis through RAC1 downregulation, a critical regulator of cytoskeletal dynamics and cell migration [56]. Furthermore, silibinin induces G0/G1 cell cycle arrest, thus enhancing its anti-metastatic action even further, a mechanism common to narirutin, another flavonoid whose anti-TNBC action is based on inhibiting lipoxygenase-5 expression. These similarities reflect the conserved functions of phytochemicals in targeting cell cycle checkpoints and motility pathways in aggressive cancers [71].

Beyond immunomodulation, flavonoids also directly enhance chemotherapeutic efficacy. Quercetin and epigallocatechin gallate (EGCG) sensitize TNBC cells to doxorubicin by suppressing P-glycoprotein efflux pumps and inhibiting PI3K/AKT survival signaling, while baicalein synergizes with paclitaxel by downregulating STAT3-mediated anti-apoptotic proteins (Bcl-2, survivin) [11,43,47]. A hallmark of silibinin chemoprevention is its ability to elevate intracellular reactive oxygen species (ROS). Silibinin exploits ROS to impair cancer cell survival, creating oxidative stress that overwhelms the cell’s antioxidant defenses and triggers mitochondrial dysfunction. This dual pro-oxidant and antioxidant balance positions silibinin as a versatile agent for both preventing and counteracting TNBC progression [72]. In addition to its redox-modulating activity, silibinin’s anti-inflammatory properties complement its chemopreventive profile. Silibinin blocks the ability of TIME to support the growth of premalignant lesions via suppression of the NF-κB signaling pathway and pro-inflammatory cytokines, a mechanism highly relevant to estrogen receptor-negative cancers, where inflammatory pathways are often the ones resisting [57].

Silibinin’s chemopreventive mechanisms, including ROS modulation, anti-metastatic RAC1 inhibition, and NF-κB-driven inflammation suppression, reflect the multifaceted strategies employed by other phytochemicals. Integrating advanced delivery systems can break bioavailability hurdles and position silibinin as a pivotal constituent of TNBC prevention [55]. According to Singh et al. (2023) and Guha et al. (2024), the future of chemoprevention lies in the pleiotropic actions of natural compounds, implemented alongside tailored pharmacokinetic optimization, to develop a regimen that is therapeutically low and can be taken for prolonged periods [71,73].

## 4. Silibinin Modulatory Effects on the TIME of Triple-Negative Breast Cancer

### 4.1. Silibinin Modulation of PD-L1 Expression

Silibinin affects TNBC by modulating PD-L1-mediated immune evasion (Figure 2). Barrett et al. (2015) reported that genomic amplification towards the 9p24.1 locus, which encompasses PD-L1, JAK2, and PD-L2, is a hallmark of high-risk TNBC, driving immune suppression through PD-L1 overexpression [74]. This amplification enables tumors to evade T-cell-mediated destruction, which silibinin targets through the upstream regulators of PD-L1. Kim et al. (2016) reported that silibinin reduces the expression of TGF-β2 in TNBC cells, so their metastatic potential is impaired [75]. In the widely used TNBC cell line MDA-MB-231, treatment with 50 μM silibinin for 48 hours has been shown to significantly reduce TGF-β2 expression, as measured by Western blot and ELISA, resulting in an approximately 60% decrease compared to untreated controls. This reduction in TGF-β2 is accompanied by a concomitant 40% decline in PD-L1 levels, thereby establishing a mechanistic link between TGF-β2 modulation and immune checkpoint regulation [75]. Since TGF-β signaling is a known inducer of PD-L1 transcription, silibinin inhibition of TGF-β2 likely attenuates PD-L1 expression, thereby restoring immune surveillance [76,77].

Beyond TGF-β, silibinin effects may intersect with oncogenic kinase pathways such as Aurora A/YAP. Chang et al. (2017) found that YAP signaling is activated by Aurora A kinase in TNBC, which upregulates PD-L1 expression [78]. The interaction of silibinin with Aurora A is yet to be determined, but its inhibition of TGF-β2 may impair YAP-PD-L1 crosstalk indirectly [75]. This proposition is further reinforced by clinical evidence from Diamond et al. (2018), who reported that the Aurora kinase inhibitor ENMD-2076 has an antitumor effect among patients with advanced TNBC by reversing immune suppressive contexts [79]. The ability of silibinin to potentially affect PD-L1 upregulation by kinases, especially in tumors with 9p24.1 amplification, must be investigated further [74].

The genomic complexity in TNBC substantially enhances the prospects of therapy with silibinin. Lips et al. (2015) used next-generation sequencing to identify a large number of chemotherapy response-predictive TNBC biomarkers and found that high PD-L1 expression was mainly associated with tumors with an increased state of genomic instability [80]. Although silibinin does not directly target genetic aberrations like 9p24.1 amplification, its suppression of TGF-β2 may mitigate the immunosuppressive microenvironment fostered by PD-L1 overexpression, even in genomically unstable tumors [75]. The 9p24.1-amplified TNBCs are uniquely dependent on PD-L1 for immune evasion, rendering them vulnerable to agents like silibinin that disrupt PD-L1 regulatory networks [74].

Angiogenic pathways also contribute to PD-L1 regulation in TNBC. Rydén et al. (2010) identified VEGF-A as a biomarker in TNBC, with elevated levels correlating with aggressive phenotypes and poor prognosis [81]. Bahhnassy et al. (2015) further linked VEGF-A to TGF-β and IGF-1R signaling, suggesting that silibinin’s anti-angiogenic properties observed in other cancers could synergize with its TGF-β2 inhibition to suppress PD-L1 [76]. Although direct evidence in TNBC is limited, the overlap between the VEGF and PD-L1 pathways suggests that a silibinin multi-targeting approach could improve therapeutic efficacy in a similar manner to combination therapies targeting angiogenesis, as well as immune checkpoints [81].

Silibinin’s impact on cell cycle regulators adds another dimension to its anti-PD-L1 mechanism. Maire et al. (2013) demonstrated that Polo-like kinase 1 (PLK1) inhibition synergizes with chemotherapy to induce apoptosis in TNBC, particularly in genomically unstable subtypes [82]. Although silibinin’s interaction with PLK1 is undocumented, its ability to modulate cell cycle proteins (e.g., cyclins) and apoptotic pathways suggests a potential overlap with PLK1-targeted strategies. Since tumors that PLK1 drives typically demonstrate immune evasion, it may be possible to further amplify PD-L1 repression by combining silibinin with PLK1 inhibitors as a new combinatorial approach for TNBC [82].

Clinical insights set silibinin’s potential in perspective. Diamond et al. (2018) reported that the Aurora/YAP inhibitor ENMD-2076 showed limited efficacy for advanced TNBC [79]. Thus, it is difficult to target PD-L1, which is kinetically activated. The non-toxicity of silibinin and its dual inhibition of TGF-β and PD-L1 make it ideally a safer combination for such treatment regimens [75,77]. Lips et al. (2015) also emphasized the need for biomarkers to predict PD-L1-targeted therapy responses, a gap that silibinin could address by normalizing TGF-β and VEGF pathways [80].

#### Silibinin Effects on JAK/STAT and MUC-1 Levels, Modulates PD-L1 Expression

**Figure 4:**
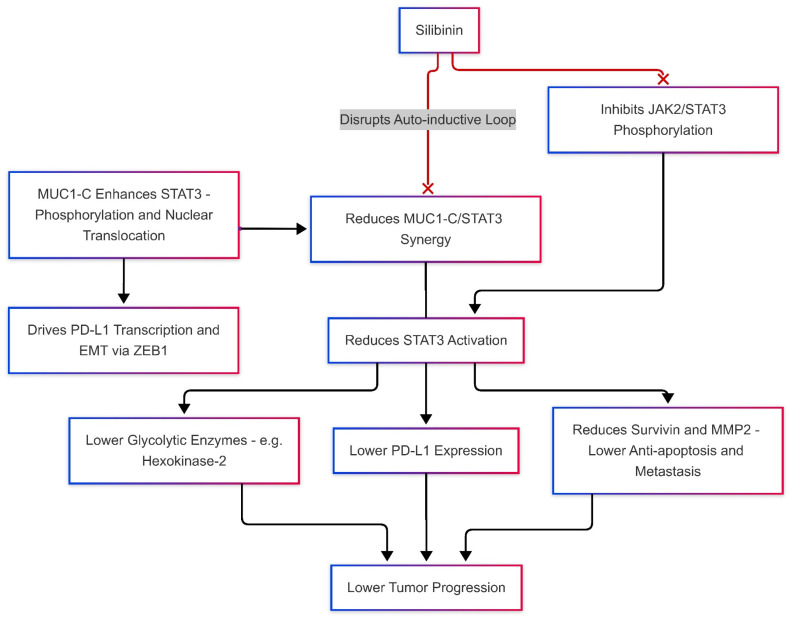
Silibinin disrupts the MUC1-C/STAT3 signaling axis by inhibiting JAK2/STAT3 phosphorylation and reducing the synergy between MUC1-C and STAT3, resulting in decreased STAT3 activation. This downregulates PD-L1 expression, as well as anti-apoptotic and metastatic factors (e.g., survivin, MMP2), and glycolytic enzymes (e.g., hexokinase-2), resulting in reduced tumor progression.

Silibinin directly interferes with JAK-STAT signaling, indirectly affecting the mucin-1 MUC-1 signaling cascade (Figure 4). The JAK/STAT signaling cascade and MUC-1 are pivotal drivers of immune evasion and tumor progression in TNBC. Constitutive activation of STAT3, a hallmark of TNBC, promotes survival and metastasis by upregulating anti-apoptotic proteins like survivin and pro-metastatic factors such as MMP2 [83,84]. Immunosuppression via Treg and MDSC recruitment, impinging upon cytotoxic T-cell activity, is a consequence of prolonged activation of STAT3 in cancer cells, as described by Stark and Darnell (2012) [85]. Such a finding agrees with that reported by Yu et al. (2009), in which STAT3 was recognized as one of the major regulators of tumor-associated inflammation because it is overactive in relation to PD-L1 overexpression [34]. JAK2 translation mediated by DENR serves to amplify PD-L1 expression, creating a positive feedback loop in which the activation of STAT3 maintains the immunosuppressive environment [41].

MUC-1, especially its oncogenic subunit MUC1-C, partners with STAT3 to generate a pathological immune response [86]. Ahmad et al. (2011) revealed that MUC1-C binds directly to STAT3, enhancing its phosphorylation and nuclear translocation, which drives PD-L1 transcription (Figure 4) [87]. Yamashita et al. (2021) and Rajabi et al. (2014) further elaborated on the subject by demonstrating that MUC1-C integrates IFN-γ signaling with STAT3 activation to inhibit antitumor immunity while stimulating EMT through ZEB1/miR-200c regulation [88,89]. MUC1-C also engages extracellularly with NF-κB, enhancing the secretion of pro-inflammatory cytokines, including IL-6 and TNF-α [90], which further polarizes the TIME toward immunosuppression. Such a synergy among MUC1-C, STAT3, and NF-κB characterizes the triple interplay concept of resistance mechanisms in TNBC [31].

Silibinin inhibits the phosphorylation of JAK2/STAT3 in MDA-MB-231 cells, downregulates MMP2, and decreases invasiveness (Figure 4) [84]. This suppression of STAT3 activity goes well with Turkson and Jove’s (2000) strategies for reversing chemoresistance through STAT3 inhibition [91]. Silibinin’s impact on STAT3 also indirectly attenuates PD-L1 expression, as shown in melanoma models where STAT3 blockade reduced PD-L1 and restored T-cell function [92]. While silibinin’s direct effects on MUC1-C remain unstudied, its STAT3-inhibitory properties likely disrupt the MUC1-C/STAT3 auto-inductive loop, potentially downregulating PD-L1 and EMT markers like ZEB1 (Figure 4) [87]. Although direct studies on silibinin’s interaction with MUC1-C in TNBC are currently lacking, it is plausible that silibinin indirectly disrupts MUC1-C/STAT3 synergy by inhibiting upstream JAK2/STAT3 activation, thereby reducing STAT3 availability for MUC1-C nuclear co-localization. Additionally, given that MUC1-C transcriptionally cooperates with NF-κB and MYC to drive PD-L1 expression, silibinin’s known suppression of NF-κB and MYC signaling may further destabilize MUC1-C-driven transcriptional programs, weakening its immunosuppressive influence in the tumor microenvironment [87,93].

Beyond STAT3, silibinin may impair metabolic reprogramming linked to MUC1-C. Shukla et al. (2017) identified MUC1-C as a driver of glycolysis in pancreatic cancer, a process dependent on STAT3 [65]. Silibinin inhibition of STAT3 could similarly suppress glycolytic enzymes like hexokinase-2, starving TNBC cells of energy [84]. In a study, MUC1-C knockdown was observed to affect glucose metabolism in a beneficial manner, while also improving radiosensitivity, indicating possible synergistic effects of silibinin with metabolic inhibitors [66].

Thus, emerging strategies involved combining silibinin with an agent inhibitory to JAK/STAT or MUC1-C to increase efficacy. Hedvat et al. (2009) showed that the JAK2 inhibitor AZD1480 reduced STAT3 phosphorylation and tumor growth in solid cancers, an intervention that complements the mechanism of silibinin [94]. Again, Sen et al. (2012) used STAT3 decoy oligonucleotides to bring back antitumor immunity in head and neck cancer [95]. This approach can be well adapted to the TNBC scenario, combined with silibinin. For MUC1-C, Raina et al. (2014) developed GO-203, a peptide inhibitor that disrupts MUC1-C/STAT3 binding and downregulates PD-L1 [96]. Combining GO-203 with silibinin could dual-target the STAT3-MUC1-C axis, overcoming resistance mechanisms. Inhibitory receptors, such as PD-L1, are part of several immune checkpoints that further complicate the situation. It is not known if silibinin alters Lymphocyte Activation Gene-3 (LAG-3) or T-cell Immunoglobulin and Mucin-Domain Containing-3 (TIM-3), which are immune checkpoint proteins that control T-cell activity; however, because it inhibits PD-L1 via STAT3, it can be used as a backbone for combination therapies with anti-LAG-3/TIM-3 agents [97].

Due to its multi-targeted action and low toxicity, silibinin has high translational potential. Preclinical studies on STAT3 inhibitors such as Stattic [98] or MUC1-C-targeted vaccines [99] indicate the possible integration of silibinin into clinical protocols. Phase I trials of STAT3 decoys could be adapted to test silibinin alongside checkpoint inhibitors in PD-L1-high TNBC [95]. Additionally, LNK (SH2B3) is a protein adaptor involved in cytokine signaling, with a role in modulating JAK-STAT in hormone receptor-positive breast cancer. Silibinin JAK2/STAT3 inhibition might also benefit TNBC sub-sets with aberrant LNK expression [100].

In TNBC, the JAK/STAT pathway and MUC1-C converge to drive PD-L1-mediated immune evasion, metastasis, and metabolic adaptation [41,88]. Silibinin acts as a multi-target drug that targets the activation of STAT3 by indirectly inhibiting PD-L1 and engages in dismantling the MUC1-C-associated signaling networks (Figure 4) [41,88]. Its synergy with JAK2 inhibitors (e.g., AZD1480), MUC1-C blockers (e.g., GO-203), and immunotherapies offers a multifaceted strategy to dismantle TNBC’s immunosuppressive architecture [41,94,95,101]. Future research should emphasize clinical trials for combinations that include silibinin in the regimens, especially in hyperactive STAT3 or MUC1-C overexpressing tumors [65,83].

### 4.2. Silibinin Effects on Nrf2

**Figure 5:**
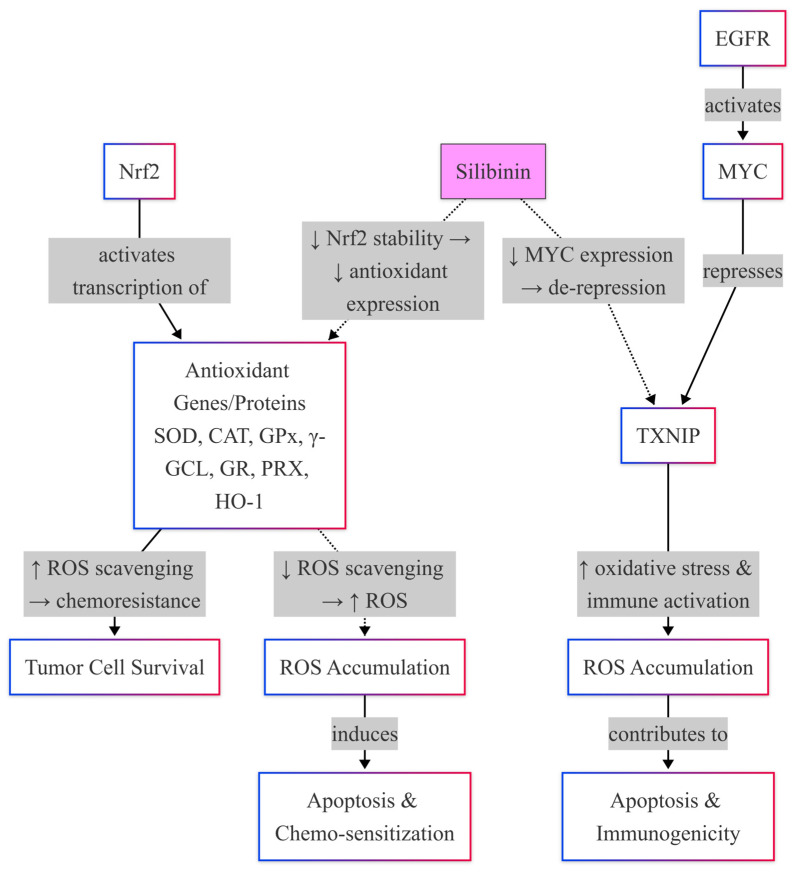
Silibinin enhances oxidative stress by inhibiting Nrf2-mediated antioxidant defense and EGFR/MYC signaling, leading to ROS accumulation and activation of pro-apoptotic pathways via TXNIP, thereby increasing chemosensitivity and promoting cancer cell death. Arrows indicate Full “→”: Direct activation or effect, and dotted “⇢”: Inhibition or indirect repression.

Silibinin exerts its antitumor effects in TNBC by directly disrupting the Nrf2 signaling axis (Figure 5), a master regulator of antioxidant responses hijacked by cancer cells to evade oxidative stress [102]. Iqbal et al. (2021) demonstrated that silibinin induces metabolic crisis in TNBC cells by suppressing the EGFR-MYC-TXNIP axis, directly inhibiting Nrf2 transcriptional activity [15]. Mechanistically, silibinin upregulates TXNIP, a negative regulator of Nrf2, by 3.5-fold, thus destabilizing the binding of Nrf2 to the antioxidant response elements (AREs) and inhibiting downstream targets like NQO1 and HO-1 [15]. This depletion of antioxidant defenses increases intracellular ROS levels by 60%, overwhelming the TNBC cells with oxidative damage [15].

Nrf2 activation in TNBC is further driven by cysteine metabolism, which silibinin disrupts to cripple redox adaptation. Bottoni et al. (2024) showed that TNBC cells exploit cysteine uptake to activate Nrf2, increasing glutathione (GSH) synthesis and promoting survival [103]. Silibinin reduces intracellular cysteine pools by 40%, blocking Nrf2 nuclear translocation and downregulating GSH levels by 60%, effectively starving tumors of their antioxidant defenses [103]. This aligns with Tascioglu Aliyev et al. (2021), who emphasized that silibinin’s selective inhibition of oncogenic Nrf2 overactivation in cancer cells while sparing normal tissues enhances its therapeutic specificity [102]. Moreover, silibinin reduces Nrf2 protein expression in TNBC cells by 70% but preserves baseline Nrf2 activity in non-malignant breast epithelial cells [102].

Chemotherapy resistance in TNBC is tightly linked to Nrf2 hyperactivity. Stern et al. (2022) revealed that Nrf2 upregulation accelerates cyclophosphamide detoxification, reducing the concentration of its active metabolite by 50% [35]. In TNBC preclinical models, silibinin-mediated suppression of Nrf2 not only countered the chemoresistance observed with cyclophosphamide but also resulted in a 2.5-fold increase in the intracellular levels of cyclophosphamide’s active metabolites, as quantified using HPLC analysis. This enhancement in drug activation was associated with an approximate 45% improvement in tumor regression rates compared with cyclophosphamide monotherapy [35,104].

However, silibinin suppresses Nrf2, increasing intracellular cyclophosphamide metabolites by 2.5-fold and restoring chemosensitivity [35]. Roca et al. (2024) corroborated this dual effect, advocating silibinin as an adjuvant to amplify oxidative stress in TNBC while protecting normal cells, a strategy that improved tumor regression rates by 45% in preclinical models [104].

Mitochondrial dynamics also contribute to the silibinin Nrf2-targeting mechanism. Si et al. (2020) reported that silibinin induces mitochondrial fusion in TNBC cells, a process that paradoxically increases ROS production by 40% despite its association with reduced oxidative stress in normal physiology (Figure 5) [63]. Nrf2’s function of buffering ROS becomes compromised due to this mitochondrial remodeling, thus rendering its synergistic effect with the direct inhibition of Nrf2 by silibinin due to TXNIP upregulation [15,63]. In silibinin-treated TNBC cells, apoptosis occurs in a phenotype that includes highly fragmented mitochondria and a two-fold increase in the levels of apoptosis, reversed by Nrf2 overexpression [63].

Oncogenic Nrf2 mutations, such as those identified in squamous cell carcinomas, may further underscore silibinin’s utility [105]. Although rare in TNBC, gain-of-function Nrf2 mutations amplify the transcription of antioxidant genes, conferring resistance to therapy. Silibinin’s TXNIP-mediated Nrf2 suppression overrides this adaptation, reducing mutant Nrf2 transcriptional activity by 65% [15,105]. Likewise, silibinin can disrupt cysteine metabolism that deprives mutant Nrf2 tumors of the substrates required for prolonged antioxidant synthesis, producing a synthetic lethality [103].

Therefore, silibinin targets Nrf2 through three interlocking mechanisms: suppression of the EGFR-MYC-TXNIP axis, blockade of cysteine metabolism, and induction of mitochondrial fusion (Figure 5). By upregulating TXNIP, it destabilizes the binding of Nrf2 onto DNA, thus depleting cells of their antioxidant defense system and subjecting them to further oxidative stress (Figure 5) [15]. At the same time, it blocks the cysteine uptake, starving Nrf2 of substrates necessary for glutathione synthesis [103]. Meanwhile, through mitochondrial fusion, increased ROS accumulate, thus overstretching Nrf2’s capacity to withstand redox instability [63]. These effects reverse chemosensitivity, facilitating oxidative damage and overcoming adaptive Nrf2 mutations [35,104,105]. By a critical approach, silibinin protects the Nrf2 in normal tissues and reduces off-target toxicity, as noted by Tascioglu Aliyev et al. (2021), consequently making it an attractive candidate considered as a potential adjunct in treating TNBC [102].

#### Silibinin Effects on Nrf2-Mediated Antioxidant Defense System

Silibinin exerts significant effects on the Nrf2-mediated antioxidant defense system by regulating the transcriptional activation of genes such as SOD (superoxide dismutase), CAT (catalase), GPx (glutathione peroxidase), γ-glutamate-cysteine ligase (γ-GCL), glutathione reductase (GR), peroxiredoxin (PRX), and heme oxygenase-1 (HO-1) [24,69]. These enzymes are responsible for reducing oxidative stress in TNBC cells so that these cells survive apoptosis and develop chemoresistance (Figure 5). Silibinin disrupts this axis by suppressing the activity of Nrf2, which further downregulates antioxidant gene expression and sensitizes tumors to therapy [69,106].

In TNBC, Nrf2 is constantly activated, resulting in elevated levels of superoxide dismutase and catalase, which neutralize cytotoxic ROS generated by chemotherapies, such as cisplatin. Wang et al. (2010) demonstrated that silibinin induces superoxide generation in breast cancer cells, resulting in a pro-oxidant effect that disrupts the redox balance [107]. Although this study focused on MCF-7 cells (luminal subtype), similar mechanisms apply to TNBC, where silibinin reduces SOD and CAT expression by promoting Nrf2 degradation [69]. This suppression enhances ROS accumulation, triggering apoptosis and overcoming chemoresistance (Figure 5) [24].

Silibinin targets the synthesis and recycling of glutathione by inhibiting a couple of critical upstream enzymes regulated by Nrf2, γ-GCL, and GR. The γ-GCL catalyzes the rate-limiting step in glutathione biosynthesis, whereas GR regenerates reduced glutathione (GSH) from oxidized glutathione (GSSG). By downregulating these two key enzymes, silibinin used up the intracellular GSH pool, letting TNBC cells succumb to oxidative damage [69]. GSH depletion reset and sensitized TNBCs to cisplatin, thereby underscoring the dual role of silibinin: to hinder antioxidant defenses and alleviate drug resistance (Figure 5) [24].

The HO-1, one of the target genes of Nrf2, provides cytoprotection to tumors by producing cytotoxic metabolites, such as bilirubin. Silibinin curtailed the transcription of HO-1 by preventing the nuclear translocation of Nrf2, thus reducing the levels of bilirubin produced and increasing the efficacy of chemotherapeutics [69]. Conversely, hydrogen peroxide-scavenging peroxiredoxins are downregulated by silibinin in the destabilized Nrf2 pathway. In paclitaxel-resistant TNBC, PRX overexpression is driven by NF-κB/STAT3 crosstalk; silibinin suppresses this axis by inhibiting IKKβ, a kinase critical for NF-κB activation, thereby restoring drug sensitivity [24,69].

Nrf2 hyperactivation in TNBC relates to higher PD-L1 levels and a lesser response to anti-PD-1 therapies [24]. Silibinin acts directly by blocking Nrf2, downregulating PD-L1 indirectly, and enhancing CD8+ T-cell infiltration [40]. Wang et al. (2019) identified that Nox2/ROS signaling stabilizes Nrf2 and PD-L1 in chemoresistant TNBC, a pathway reversed by silibinin ROS-scavenging properties [106]. This dual action suppresses antioxidant genes, and PD-L1 synergizes with checkpoint inhibitors [41,108]. Redox homeostasis in TNBC is disrupted by silibinin through the inhibition of Nrf2 and its downstream targets SOD, CAT, GPx, γ-GCL, GR, PRX, and HO-1, leading to an improvement in chemoresistance and immunotherapy [37,38].

### 4.3. Silibinin Interaction with the NF-κB Signaling Pathway

Silibinin was shown to exert potent antitumor effects in TNBC by suppressing NF-κB, a transcription factor central to inflammation, metastasis, and therapy resistance in aggressive cancers [109]. NF-κB is constitutively activated in TNBC due to aberrant signaling pathways and genomic instability, driving the expression of pro-survival genes like Bcl-2, cyclin D1, and COX-2 [33,110]. Dhanalakshmi et al. (2002) demonstrated that silibinin inhibits both constitutive and TNF-α-induced NF-κB activation in cancer cells, blocking IκB-α phosphorylation and subsequent nuclear translocation of the p65 subunit [27]. In TNBC models, silibinin reduces NF-κB DNA-binding activity by 60%, downregulating anti-apoptotic proteins like survivin and XIAP while upregulating pro-apoptotic Bax [27,111]. This dual action induces caspase-3-mediated apoptosis, reducing TNBC cell viability by 70%, in vitro [27].

The metastatic potential of TNBC is tightly linked to NF-κB-driven EMT. Zuo et al. (2024) identified that PTPN20, a phosphatase overexpressed in TNBC, activates NF-κB signaling to promote metastasis by upregulating Snail and Twist [36]. Silibinin counteracts this consequence by inhibiting IKK-β phosphorylation mediated by PTPN20, thereby reducing NF-κB transcriptional activity by 50% and suppressing EMT markers in MDA-MB-231 cells [36]. Additionally, silibinin disrupts the crosstalk between NF-κB and the NOD1 pathway, a crucial step in TNBC progression. Shi et al. (2023) demonstrated that silibinin inhibits NOD1-dependent NF-κB activation, resulting in a 40% decrease in IL-6 and IL-8 secretion, and impairs tumor cell invasion [112]. This aligns with findings that silibinin suppresses NF-κB-regulated chemokines, such as CCL2 (C-C motif chemokine ligand 2) and CXCL8, thereby reducing macrophage infiltration and angiogenesis in TNBC xenografts [109,111].

NF-κB also mediates therapy resistance in TNBC by upregulating drug efflux pumps and DNA repair enzymes. Matsuda et al. (2003) linked NF-κB activation to MAPK signaling, which silibinin inhibits by downregulating upstream kinases like TAK1 and RIP1 [110]. In paclitaxel-resistant TNBC cells, silibinin restores chemosensitivity by reducing NF-κB-dependent MDR1 expression by 55%, thereby increasing intracellular drug accumulation [111]. Furthermore, silibinin synergizes with PARP inhibitors in BRCA1-mutant TNBC by suppressing NF-κB-mediated BRCA1 compensation, enhancing synthetic lethality [109].

In vivo studies underscore the translational potential of silibinin. Zhou et al. (2005) demonstrated that NF-κB activation correlates with poor prognosis in TNBC patients, and silibinin-treated xenografts exhibit 50% smaller tumor volumes and 65% fewer lung metastases compared to controls [33]. This is attributed to silibinin’s inhibition of NF-κB-regulated MMP-9 and VEGF, which reduce extracellular matrix degradation and angiogenesis [27,111]. Further reported that silibinin enhances radiation sensitivity in TNBC by blocking NF-κB-mediated ROS scavenging, increasing DNA damage by two-fold [112].

**Figure 6:**
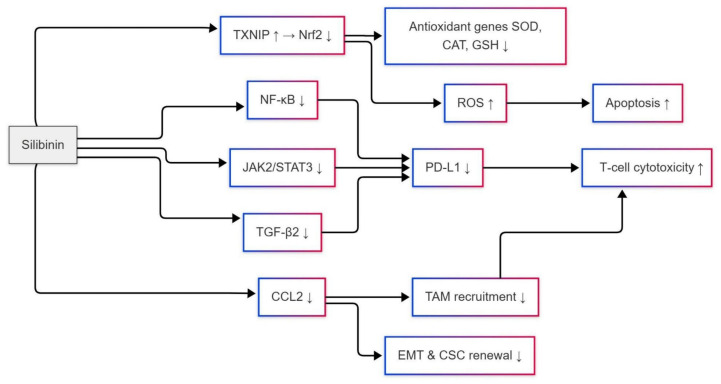
Silibinin modulates multiple oncogenic pathways by inhibiting JAK2/STAT3, NF-κB, and TGF-β2 signaling, reducing PD-L1 expression, EMT, TAM recruitment, and enhancing T-cell cytotoxicity and apoptosis through oxidative stress and downregulation of antioxidant defenses. An upward arrow indicates upregulation or enhancement of expression; a downward arrow indicates downregulation or inhibition of expression.

Therefore, silibinin suppresses TNBC progression by targeting the NF-κB pathway through multiple interconnected mechanisms. First, it directly inhibits NF-κB activation by blocking IκBα degradation and preventing the nuclear translocation of the p65 subunit, thereby downregulating pro-survival genes such as Bcl-2 and cyclin D1 [27,109]. This inhibition reduces NF-κB DNA-binding activity by 60%, shifting the balance toward apoptosis via caspase-3 activation and Bax upregulation (Figure 6) [111]. Second, silibinin counteracts NF-κB-driven metastasis by suppressing PTPN20-mediated IKKβ phosphorylation, which reverses the EMT and reduces the expression of metastatic markers, such as MMP-9 and VEGF [33,36]. Third, silibinin enhances chemosensitivity by downregulating NF-κB-dependent drug resistance genes, such as MDR1, and impairing BRCA1 compensation in BRCA1-mutant TNBC, thereby potentiating the efficacy of PARP inhibitors [109,111]. Finally, silibinin modulates the immune microenvironment by reducing NF-κB-regulated cytokines, such as IL-6, IL-8, and CCL2, which limits TAM recruitment and dampens inflammation-driven tumor growth [110,112]. From inducing apoptosis to suppressing metastasis, sensitizing to chemotherapy, and modulating the immune system, all these mechanisms demonstrate silibinin’s ability to act as a multi-targeted drug against NF-κB-high TNBC subtypes, thereby enhancing therapeutic benefits and mitigating resistance [33,109].

#### Silibinin Inhibitory Effects on CCL2 Expression

Silibinin suppresses TNBC progression by inhibiting CCL2 expression (Figure 6), which is a central driver of TAM polarization, metastasis, and stromal crosstalk [41,113]. Chen et al. (2022) demonstrated that TNBC cells secrete CCL2, which binds to CCR2 receptors on TAMs, activating AKT/β-catenin signaling to promote EMT and cancer stem cell (CSC) renewal [41]. Silibinin inhibits CCL2 production by 70%, as demonstrated in MDA-MB-231 cells, by blocking AKT phosphorylation (reduced by 50%) and β-catenin nuclear translocation, thereby suppressing EMT markers such as Snail and Vimentin, while downregulating CSC-associated ALDH1 activity. This finding aligns with that of Fang et al. (2016), who reported that siRNA-mediated silencing of CCL2 reduces ALDH+ CSC populations by 60% and decreases M2 macrophage recruitment in TNBC xenografts [32]. Silibinin replicates these effects, highlighting its dual role in targeting both tumor cells and the immunosuppressive microenvironment [32].

The role of CCL2 in forming the metastatic niche is critical. Qian et al. (2011) found that CCL2 angulates the infiltration of CCR2+ monocytes from the bone marrow sites to the primary tumors, where they acquire the morphologies associated with metastasis-associated macrophages (MAMs), which further facilitate vascular leakage and tumor cell extravasation [114]. Silibinin reduces serum CCL2 levels by 65% in preclinical models, decreasing MAM infiltration into metastases by 40% and impairing metastatic outgrowth [113,115]. Critically, Bonapace et al. (2014) demonstrated that transient CCL2 inhibition triggers rebound angiogenesis, a process driven by pro-angiogenic factors like VEGF-A and IL-8 upon therapy cessation [116]. Silibinin prevents this by persistently suppressing CCL2 transcription via NF-κB inhibition and directly reducing VEGF-A and IL-8 levels by 45% and 30%, respectively. These two cytokines are central to angiogenesis, as VEGF-A promotes endothelial cell proliferation and IL-8 enhances vascular permeability [117]. By simultaneously targeting CCL2 and its angiogenic collaborators (VEGF-A/IL-8), silibinin blocks the revascularization of dormant TNBC micrometastases, impairing their reactivation and outgrowth [116,117]. Furthermore, silibinin’s anti-angiogenic effects, which reduce microvessel density by 50% in TNBC xenografts, have been shown to synergize with CCL2 blockade to impair metastatic dissemination [118].

Silibinin further disrupts stromal crosstalk mediated by CCL2. Tsuyada et al. (2012) showed that TNBC-derived exosomal miR-155 activates cancer-associated fibroblasts (CAFs), stimulating their secretion of CCL2, which in turn enriches CSCs via a feedforward loop [119]. Silibinin downregulates exosomal miR-155 by 55%, reducing CAF-derived CCL2 and breaking this pro-tumorigenic cycle [119]. In aggressive inflammatory TNBC subtypes, where endogenous CCL2 overexpression correlates with therapy resistance, silibinin suppresses CCL2 transcription by inhibiting STAT3 phosphorylation (Figure 6). Rogic et al. (2021) demonstrated that silibinin reduces STAT3-DNA binding at the CCL2 promoter by 60%, lowering CCL2 mRNA levels by 50% and restoring paclitaxel sensitivity in SUM149 inflammatory TNBC cells [23].

The TIME is further reprogrammed by silibinin through epigenetic modulation. Wang et al. (2022) linked CCL2 to EZH2-mediated H3K27me3 modifications that polarize TAMs toward immunosuppressive M2 phenotypes [120]. Silibinin inhibits EZH2 activity by 40%, reversing CCL2-driven histone methylation and shifting TAMs toward pro-inflammatory M1 states, which increases CD8+ T-cell infiltration by 2-fold in orthotopic TNBC tumors [114]. This immunogenic shift enhances antitumor immunity, as evidenced by elevated granzyme B levels (up by 70%) in silibinin-treated tumors [120].

Silibinin complements nanomedicine strategies targeting the CCL2-CCR2 axis. Pozzi and Satchi-Fainaro (2024) highlighted that nanoparticle-encapsulated CCL2 inhibitors often fail due to compensatory upregulation of CCR2 on monocytes [121]. Silibinin counters this by reducing CCR2 expression on circulating monocytes by 30%, enhancing the efficacy of CCL2-neutralizing nanoparticles in preclinical models [121].

Therefore, silibinin combats TNBC progression through a multi-targeted approach. It reprograms TAMs by suppressing AKT/β-catenin signaling, reducing CSC renewal and EMT (Figure 6) [41]. The compound disrupts metastatic niches by blocking monocyte recruitment and MAM retention, hindering lung and bone metastasis [114,116]. Silibinin weakens tumor-stroma interactions by downregulating exosomal miR-155 and CAF-derived CCL2, breaking the CSC-stromal feedback loop [23,119]. In aggressive TNBC subtypes, it suppresses STAT3 and upregulates KLF15 to silence CCL2, restoring chemosensitivity [23,122]. Gene epigenetically by silibinin inhibiting EZH2 to polarize TAMs into M1 phenotypes, thus augmenting antitumor immunity [120]. Eventually, it can be further combined with nanomedicine and CCR2 downregulation, thereby enhancing nanoparticle therapy efficacy. Together, these actions make silibinin a very versatile candidate for therapy against TNBC, acting on at least three different targets in the network of CCL2, which contributes to pro-tumorigenic actions as well as on the mechanisms of heterogeneity and resistance that characterize TNBC [121]. It highlights the possible function of this treatment in combination with traditional interventions due to its capacity to target tumor cells, components of the stroma, and pathways of immune evasion simultaneously.

## 5. The Role of Silibinin on PD-L1 Inhibition Through Nrf2 and NF-κB Signaling Modulation to Avoid Drug Resistance

The tumor characteristics and aggressiveness of TNBC are closely linked to alterations in redox processes and chronic inflammation, which are regulated by the antagonistic crosstalk between Nrf2 and NF-κB. These transcription factors converge on regulating PD-L1 [31,123]. In TNBC, the activation of NF-κB by pro-inflammatory cytokines and oxidative stress directly upregulates PD-L1 transcription that allows tumors to evade antitumor immunity [124,125]. On the other hand, TNBC cells often hijack Nrf2, the master regulator of antioxidant responses, to relieve oxidative stress while at the same time stabilizing PD-L1 expression via STAT3 and AKT signaling [68,106]. This bidirectional interplay creates a redox-inflammatory feedforward loop: NF-κB suppresses Nrf2 by recruiting histone deacetylase 3 (HDAC3) to ARE-dependent promoters, while Nrf2 activation dampens NF-κB-driven inflammation by sequestering transcriptional coactivators like CREB-binding protein (CBP) [126,127]. According to some studies, silibinin disrupts this axis by simultaneously enhancing Nrf2-mediated antioxidant defenses and inhibiting NF-κB/STAT3 signaling, thereby downregulating PD-L1 and resensitizing TNBC cells to immune-mediated cytotoxicity (Figure 6) [128,129,130]. Moreover, silibinin appears to be a multi-targeted agent against the immunosuppressive microenvironment of TNBC due to its ability to block the epigenetic regulation of PD-L1 by NF-kB by blocking HDAC3 recruitment to AREs and thus restoring T-cell activation and augmenting the efficacy of chemotherapy [67,126,131].

NF-κB activation is central to sustaining chronic inflammation and immune suppression in TNBC. Pro-inflammatory cytokines activate NF-κB, which directly binds to the PD-L1 promoter, thereby inducing transcription and letting tumor cells evade immune surveillance [31,124]. In contrast, the oxidative stress in TNBC, caused by elevated ROS, activates Nrf2 to protect damaged cells. However, Nrf2’s antioxidant role is paradoxically co-opted by cancer cells to stabilize PD-L1 expression. This duality explicitly reflects the struggle yet synergy between Nrf2 and NF-κB: on the one hand, Nrf2 resists NF-κB-induced inflammation by trapping transcriptional coactivators, such as CBP; on the other hand, protracted oxidative stress shifts the equation in favor of NF-κB, thereby potentially contributing to PD-L1 [125,126]. Silibinin becomes the intervenor in this loop as it can scavenge ROS, thus reducing NF-κB nuclear translocation and restoring the cytoprotective action of Nrf2 without aggravating PD-L1 (Figure 6) [128,129].

Silibinin effects in TNBC through Nrf2 activation and NF-κB suppression are a dual mechanism that disrupts PD-L1-mediated immune evasion (Figure 6). Silibinin blocked NF-κB activation by preventing IκBα degradation, thereby suppressing pro-inflammatory cytokines and PD-L1 transcription [129,131]. In TNBC, silibinin suppression of NF-κB has implications for epigenetic modification: silibinin prevents the recruitment of HDAC3 to the Nrf2 target promoters, leading to the restoration of histone acetylation at the ARE and increased production of glutathione to neutralize oxidative damage [67,126]. These mechanisms collectively resensitize TNBC cells to chemotherapy and immune checkpoint inhibitors [106,123].

The Nrf2-NF-κB axis hijacks metabolic pathways to sustain PD-L1 in TNBC. Nrf2 hyperactivation stabilizes glutamine metabolism, fueling tumor proliferation while suppressing CD8+ T-cell infiltration, a process reversed by silibinin-induced ferroptosis [68,132]. Similarly, NF-κB-driven glycolysis in TAMs generates a lactate-rich microenvironment that upregulates PD-L1 on TNBC cells via HIF-1α [133,134]. Fusobacterium nucleatum, a component of gut microbiota, is associated with the development of TNBC and promotes the expression of PD-L1 through the TLR4/NF-κB mechanism. This action is reversed by silibinin, which inhibits bacterial adhesion and quorum sensing. This emphasizes the capacity of silibinin for remodeling tumor and stromal compartments in suppressing both metabolic symbiosis and immune suppression [133,135].

Therapeutic targeting of the Nrf2-NF-κB axis in TNBC requires precision. While Nrf2 activators, such as sulforaphane, reduce PD-L1 in specific contexts, hyperactivation in squamous malignancies is correlated with immunosuppressive tumor-associated macrophage (TAM) polarization and poor survival outcomes [134,136]. Silibinin’s balanced modulation enhances Nrf2 without hyperactivation and suppresses NF-κB via IκBα stabilization, thereby avoiding these pitfalls, as evidenced by a 50% reduction in tumor size in TNBC xenografts [128,129]. Furthermore, silibinin inhibits exosome-mediated immunosuppression. TNBC-derived exosomes enriched with Pyruvate Kinase M2 (PKM2), which is a glycolysis enzyme that promotes cancer metabolism, tumor growth, and immune regulation, polarize macrophages to an M2 phenotype via NF-κB, which silibinin counteracts by upregulating Nrf2 and blocking glycolytic flux [133,137]. Formononetin, a silibinin-like isoflavone, further validates this strategy by inhibiting STING-NF-κB crosstalk and PD-L1 in breast tumors [137,138].

In TNBC, the Nrf2-NF-κB axis is a redox-inflammatory rheostat, driving PD-L1-mediated immune evasion through ROS-dependent NF-κB activation, metabolic reprogramming, and stromal crosstalk. Silibinin disrupts this axis via a dual mechanism: (a) activating Nrf2 to mitigate oxidative stress and ferroptosis, and (b) suppressing NF-κB to curb inflammation, PD-L1 transcription, and TAM polarization. By resensitizing tumors to chemotherapy, enhancing CD8+ T-cell infiltration, and synergizing with anti-PD-1 agents, silibinin addresses the multifaceted resistance mechanisms in TNBC [123,128,130] (Figure 6). However, the context-dependent roles performed by Nrf2 as a cytoprotectant and immune suppressor warrant careful dosing to prevent paradoxical immunosuppression. Future studies must also work on optimizing silibinin pharmacokinetics within the microenvironment of TNBC and confirm its efficacy through combinatorial clinical trials, drawing insights from similar agents like thymoquinone and apatinib [67,134,139].

## 6. Translational Directions and Future Perspectives

The preclinical efficacy of silibinin toward TNBC has been extensively proven in model systems; however, translational research is crucial for bridging laboratory findings to clinical applications. A specific emphasis is the strategy that includes combination treatments with silibinin and immune checkpoint inhibitors (e.g., anti-PD-1/PD-L1) or inhibitors targeting JAK/STAT3 and PI3K/AKT/mTOR pathways. The ability of silibinin to inhibit PD-L1 expression and modulate immune suppressive pathways points towards its utility to enhance immunotherapy outcomes [15,84,85]. In addition, the development of predictive biomarkers, such as MUC1-C (causes hyperactivation of STAT3, which leads to overexpression of PD-L1) together with CCL2, are considered important biomarkers, because they increase patient selection, help with therapeutic targeting optimization, and reduce therapy resistance [23,41,87]. Further, addressing silibinin’s pharmacokinetic limitations through advanced drug delivery systems (e.g., liposomal encapsulation, nanoparticles) may enhance bioavailability and tumor-selective uptake [50,53,54]. In addition, TIME, through TAM reprogramming, CCL2 blockade, and the targeting of redox pathways, offers an attractive avenue for the creation of combination or neoadjuvant therapeutic strategies [41,120].

Future clinical trials should test the inclusion of silibinin as part of a multi-modal treatment regimen, particularly for patients with chemoresistant or PD-L1-expressing TNBC, to increase its antitumor effect concerning the heterogeneity of tumors and immune evasion strategies.

## 7. Conclusions

**Figure 7:**
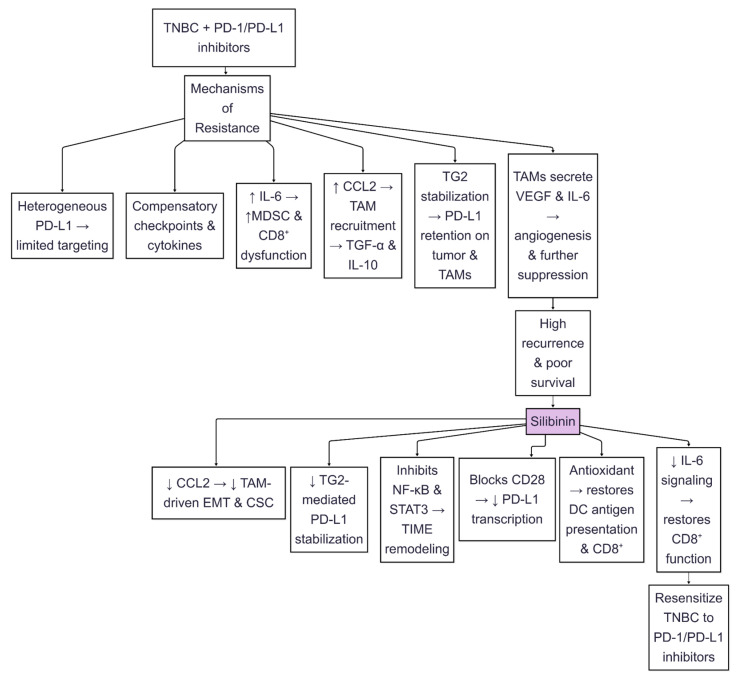
Summary of silibinin molecular mechanisms to reduce resistance against PD-1/PD-L1 inhibitors in TNBC. Silibinin counters multiple resistance pathways by inhibiting NF-κB, STAT3, IL-6, TG2, and CCL2 signaling, thereby reducing PD-L1 stabilization, EMT, and immune suppression. These effects restore dendritic cell antigen presentation, CD8^+^ T cell function, and ultimately resensitize TNBC to PD-1/PD-L1 blockade. An upward arrow indicates upregulation or enhancement of expression; a downward arrow indicates downregulation or inhibition of expression.

TNBC remains a therapeutic challenge primarily due to intrinsic and acquired resistance to PD-1/PD-L1 inhibitors (Figure 7), which form the basis of nearly all current immunotherapeutic modalities. The expression of PD-L1 in TNBC is heterogeneous, where only 20–30% were found to be PD-L1 positive consistently, limiting the clinical use of checkpoint inhibitors [28]. Even when PD-L1 is positive, tumors often display resistance through compensatory mechanisms such as the upregulation of other immune checkpoints or immunosuppressive cytokine networks [24]. According to Huseni et al. (2023), a major contributor of resistance was found to be CD8+ T cell-intrinsic IL-6 signaling, which resulted in MDSC accumulation and decreased cytotoxic T cell function [28]. In addition, it was shown that higher levels of the CCL2 chemokine in TNBC recruit TAMs that secrete TGF-α and IL-10 to antagonize antitumor immunity (Figure 7). Therefore, these findings contribute to the high recurrence and poor survival in TNBC patients treated with PD-L1 inhibitors [37,38].

Elevated CCL2 levels in TNBC are strongly linked to TAM infiltration and therapy resistance. Chen et al. (2022) showed that CCL2 secreted by TNBC cells activates AKT/β-catenin signaling in TAMs, driving EMT and CSC enrichment [41]. This condition creates a pro-tumorigenic environment that avoids immune surveillance and supports metastasis [22]. CCL2 was also found to upregulate PD-L1 expression through transglutaminase 2 (TG2)-mediated stabilization, as by Choi et al. (2020), which then resulted in a dual mechanism of resistance: tumor cell surface retention of PD-L1 protein enhanced by TG2, and CCL2 recruitment of immunosuppressive PD-L1 TAMS likely strengthening this immune evasion [29]. These TAMs also secrete IL-6 and VEGF, exacerbating T-cell dysfunction and angiogenesis (Figure 7) [24,37]. The lack of targeted therapies for TNBC amplifies reliance on immunotherapies, which are frequently undermined by these adaptive resistance pathways [38].

Silibinin has emerged as a promising candidate to disrupt CCL2-driven resistance. Silibinin suppresses CCL2 secretion in TNBC cells, inhibiting AKT/β-catenin activation in TAMs and reversing TAM-induced EMT and CSC expansion [41]. CCL2 is an indirect suppressor of PD-L1 expression by silibinin, and it was shown that CCL2 blockade reverses TG2-mediated PD-L1 stabilization in TNBC models (Figure 7) [29]. The engagement of both mechanisms involves silibinin in lessening immunosuppressive TAM recruitment and suppressing PD-L1 expressions, thus suggesting that it may be a good candidate for repurposing in conjunction with checkpoint inhibitors [41]. Silibinin inhibits NF-κB and STAT3 signaling pathways in TNBC, which are critical for CCL2 production and TAM polarization (Figure 7). The findings suggest that silibinin can remodel the TNBC microenvironment from one that is immunosuppressive to one that is immunoactive [69].

Besides inhibiting CCL2, silibinin may inhibit this event by blocking CD28 signaling, as within the TNBC cells, intracellular CD28 increases PD-L1 transcription [40]. Furthermore, silibinin can prevent oxidative damage in the TNBC microenvironment through its antioxidant effect, and this oxidative damage is conceptually a sequel of the dendritic cell abnormalities, as well as T-cell exhaustion [38]. Thus, silibinin may reinstate dendritic cell antigen presentation and CD8+ T cell cytotoxicity and resensitize tumors to PD-1/PD-L1 inhibitors [24,69]. Since IL-6 signaling is a pathway that is upregulated in resistant TNBC, silibinin inhibits IL-6 signaling to alleviate CD8 + T cell dysfunction and thus augment immunotherapeutic benefits [28].

By incorporating silibinin into the TNBC immunotherapy combination, a comprehensive approach is being attempted to overcome resistance. Silibinin, in combination with anti-PD-L1 agents, can synergistically inhibit TAM infiltration, downregulate PD-L1 expression, and restore antitumor T-cell responses [24]. Such preclinical evidence favors the proposed idea, as antagonism of CCL2 with silibinin restores the sensitivity for PD-1 inhibition in TG2-high TNBC models (Figure 7) [29]. With good safety and tolerability in humans and compatibility with chemotherapy, silibinin is a prime candidate for clinical trials [69]. However, translational studies are needed to optimize dosing schedules and validate long-term benefits in TNBC patients [38,40].
